# Supplementary material for: Dual stimulation of CD40 and 41BB pathways during ex-vivo TIL expansion enhances CD8+ T cell expansion
Source: bioRxiv. 2026 Jul 26:2026.07.22.740128. Preprint. [Version 1] doi: 10.64898/2026.07.22.740128 (PMC13419458; doi:10.64898/2026.07.22.740128)
Supplement: Supplement 1 [file media-1.pdf]

Supplemental Table 1. Antibodies for the flow cytometry assays.

Antibody	Fluorochrome	Clone	Titer	Manufacturer	Catalog #
**41BB**	BV421	4B4-1	1:50	Biolegend	309820
**CD3**	BUV395	SK7	1:100	BD	564001
**His-Tag**	Alexa Fluor 647	-	1:50	Cell Signaling	14931S
**CD4**	BUV496	SK3	1:100	BD	612936
**CD4**	BV785	OKT4	1:25	Biolegend	317442
**CD8**	PECy7	RPA-T8	1:400	BD	557746
**CD19**	BV605	HIB19	1:100	Biolegend	302244
**CD27**	BV605	O323	1:100	Biolegend	302830
**CD39**	FITC	A1	1:200	Biolegend	328206
**CD40**	BV786	5C3	1:100	Biolegend	334340
**CD56**	PE	NCAM-1	1:50	BD	555516
**CD62L**	BV421	DREG-56	1:50	Biolegend	304828
**CD69**	APC	FN50	1:25	BD	555533
**CD80**	BV650	2D10	1:25	Biolegend	305227
**CD86**	PECy7	IT2.2	1:400	Biolegend	305422
**HLA-DR**	FITC	Tu39	1:400	BD	555558
**PD-1**	Alexa Fluor 700	EH12.2H7	1:50	Biolegend	329952
**TIM-3**	BV650	7D3	1:100	BD	565564
**Live/Dead**	Near IR	-	1:500	Invitrogen	L10119

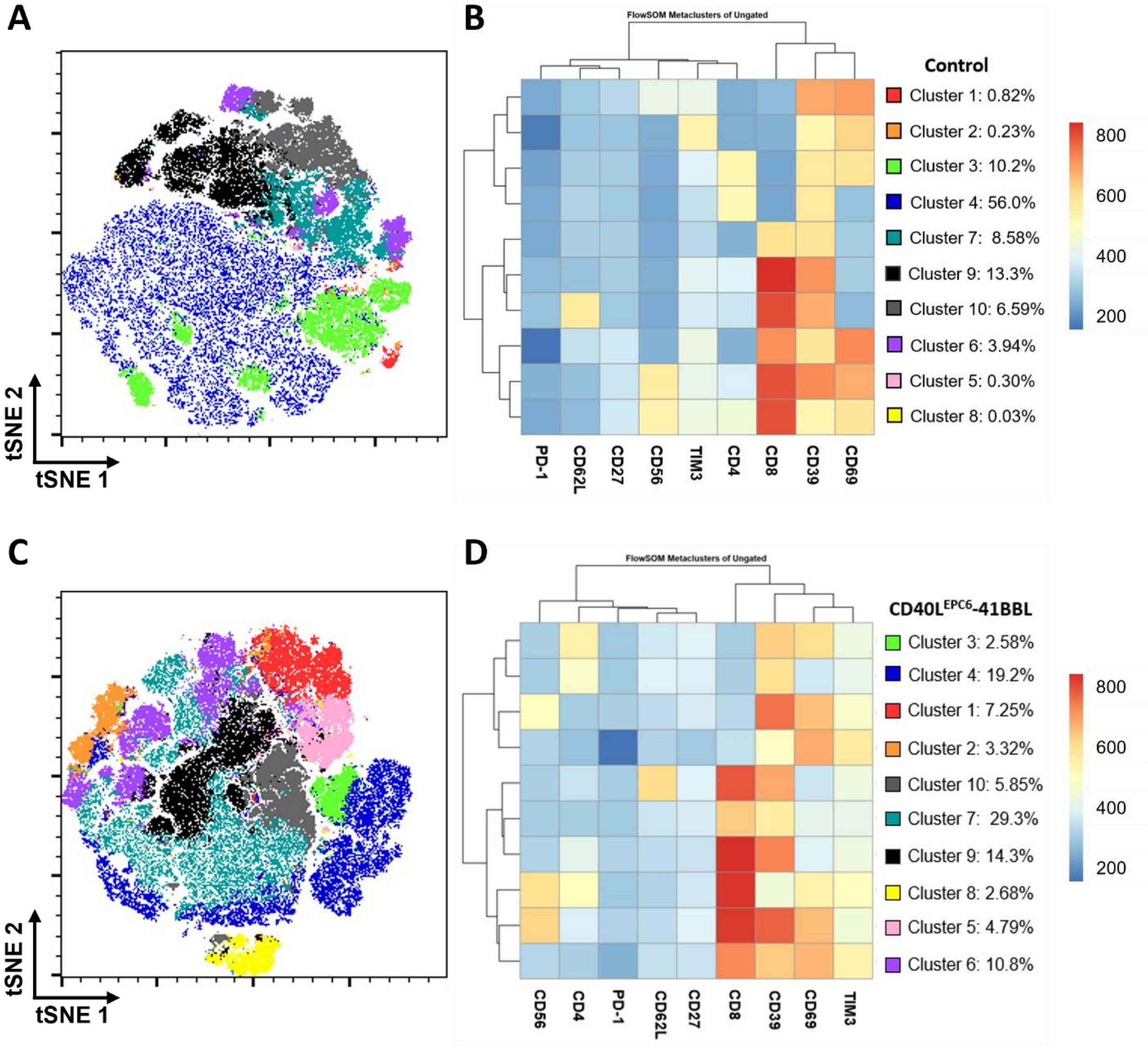

E

**CD4- CD8-**	Clusters: **1**, 2.
**CD4+ CD8-**	Clusters: **3**, **4**.
**CD4- CD8+**	Clusters: **5**, **6**, 7, **9**, 10.
**CD4+ CD8+**	Clusters: 8.

Supplemental Figure 1. High-dimensional flow cytometry analysis with FlowSOM clustering in lung tumor samples. FlowSOM-generated tSNE maps and metacluster heatmaps for each culture condition. Lung tumor fragments were cultured for 3–4 weeks

in **(A-B)** standard TIL expansion media (Control, IL-2 only) or **(C-D)** media supplemented with 0.043 uM CD40L^{EPC6}-4BBL (n=10). **(E)** CD4 and CD8 expression cluster distribution.

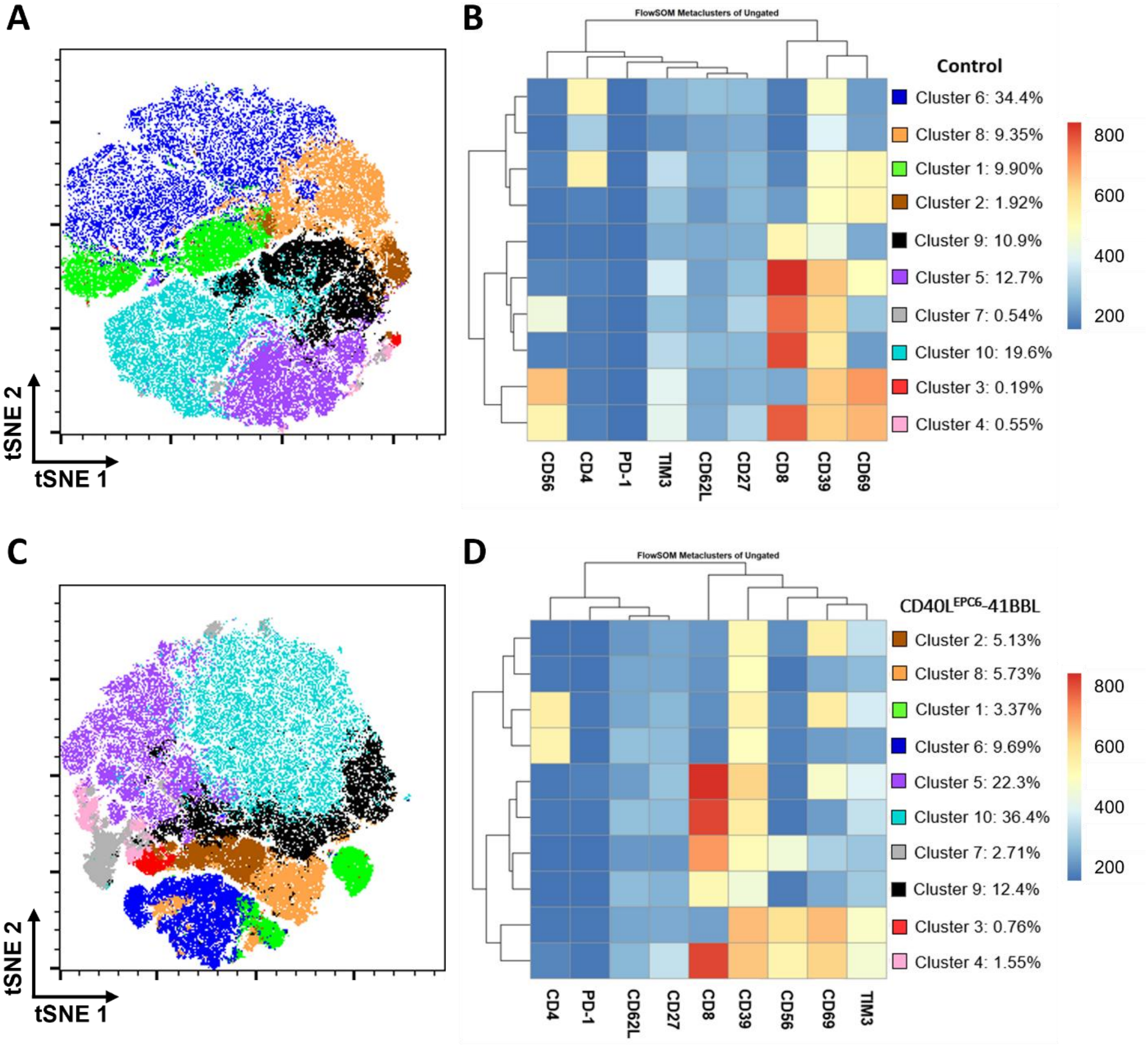

E

**CD4- CD8-**	Clusters: 2, **3**, 8.
**CD4+ CD8-**	Clusters: **1**, **6.**
**CD4- CD8+**	Clusters: **4**, **5**, 7, **9**, 10.
**CD4+ CD8+**	Clusters: none.

Supplemental Figure 2. High-dimensional flow cytometry analysis with FlowSOM clustering in melanoma samples. FlowSOM-generated tSNE maps and metacluster heatmaps for each culture condition. TIL. Melanoma fragments were cultured for 3–4

weeks in **(A-B)** standard TIL expansion media (Control, IL-2 only) or **(C-D)** media supplemented with 0.043 uM CD40L^{EPC6}-4BBL (n=9). **(E)** CD4 and CD8 expression cluster distribution.

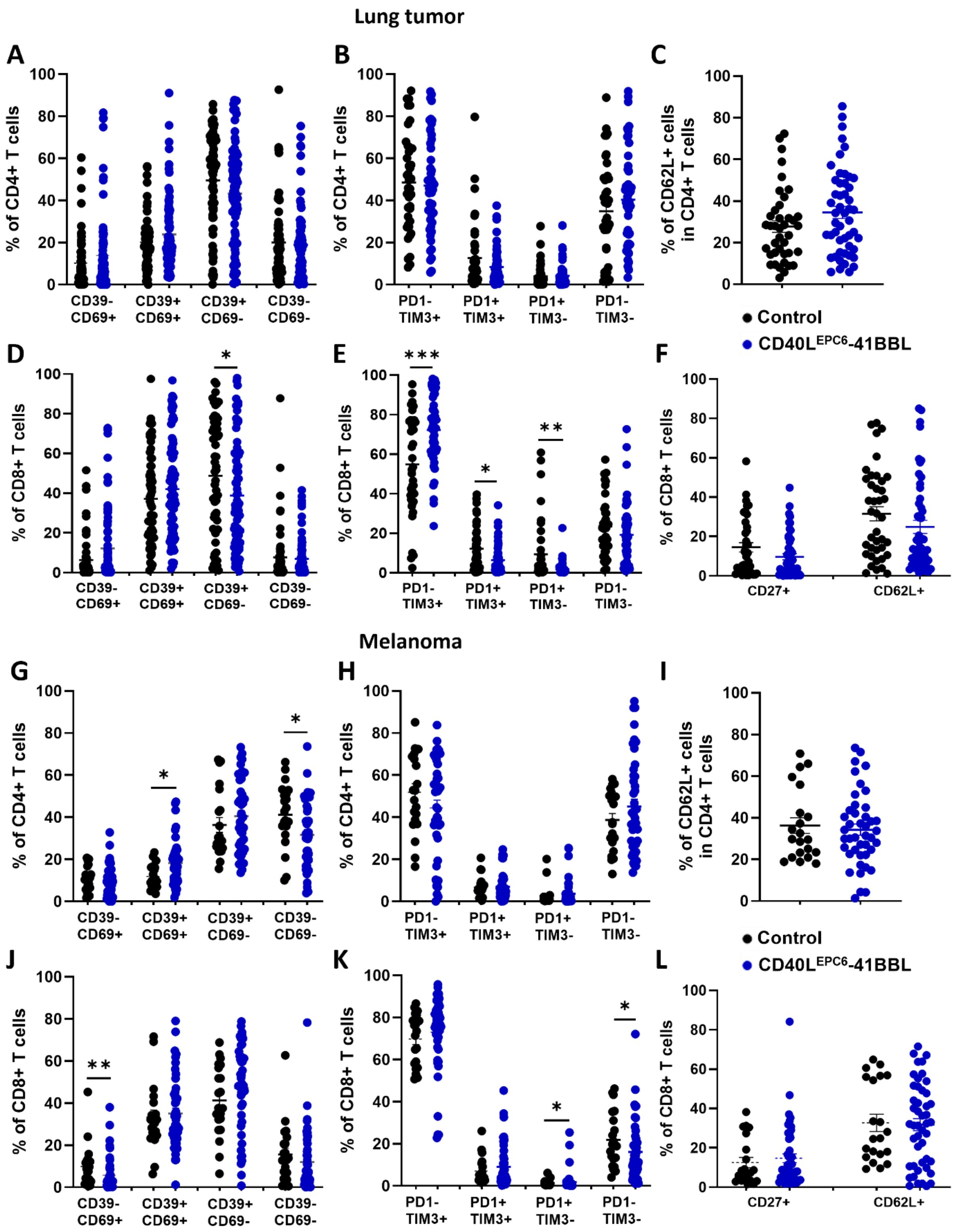

Supplemental Figure 3. Conventional flow cytometry analysis in lung tumor and melanoma samples. Fragments were cultured for 3–4 weeks in standard TIL expansion

media (Control) or media supplemented with 0.043 μM CD40L^{EPC6}-4BBL **(A-F)** Percentage of CD4^{+} and CD8^{+} T cells subpopulations in expanded TIL from lung tumor fragments. **(G-L)** Percentage of CD4^{+} and CD8^{+} T cells subpopulations in expanded TIL from melanoma fragments. *p ≤ 0.05, **p ≤ 0.01, ***p ≤ 0.001.
